# The burden of malaria in post-emergency refugee sites: A retrospective study

**DOI:** 10.1186/1752-1505-5-17

**Published:** 2011-09-19

**Authors:** Jamie Anderson, Shannon Doocy, Christopher Haskew, Paul Spiegel, William J Moss

**Affiliations:** 1 Bloomberg School of Public Health, Johns Hopkins University, Baltimore, Maryland, USA; 2 United Nations High Commissioner for Refugees, Geneva, Switzerland

## Abstract

**Background:**

Almost two-thirds of refugees, internally displaced persons, returnees and other persons affected by humanitarian emergencies live in malaria endemic regions. Malaria remains a significant threat to the health of these populations.

**Methods:**

Data on malaria incidence and mortality were analyzed from January 2006 to December 2009 from the United Nations High Commissioner for Refugees Health Information System database collected at sites in Burundi, Chad, Cameroon, Ethiopia, Kenya, Sudan, Tanzania, Thailand, and Uganda. Data from three countries during 2006 and 2007, and all nine countries from 2008 to 2009, were used to describe trends in malaria incidence and mortality. Monthly counts of malaria morbidity and mortality were aggregated into an annual country rate averaged over the study period.

**Results:**

An average of 1.18 million refugees resided in 60 refugee sites within nine countries with at least 50 cases of malaria per 1000 refugees during the study period 2008-2009. The highest incidence of malaria was in refugee sites in Tanzania, where the annual incidence of malaria was 399 confirmed cases per 1,000 refugees and 728 confirmed cases per 1,000 refugee children younger than five years. Malaria incidence in children younger than five years of age, based on the sum of confirmed and suspected cases, declined substantially at sites in two countries between 2006 and 2009, but a slight increase was reported at sites within four of seven countries between 2008 and 2009. Annual malaria mortality rates were highest in sites in Sudan (0.9 deaths per 1,000 refugees), Uganda and Tanzania (0.7 deaths per 1000 refugees each). Malaria was the cause of 16% of deaths in refugee children younger than five years of age in all study sites.

**Conclusions:**

These findings represent one of the most extensive reports on malaria among refugees in post-emergency sites. Despite declines in malaria incidence among refugees in several countries, malaria remains a significant cause of mortality among children younger than five years of age. Further progress in malaria control, both within and outside of post-emergency sites, is necessary to further reduce malaria incidence and mortality among refugees and achieve global goals in malaria control and elimination.

## Background

All-cause mortality rates in refugee populations living in camps have decreased since the 1990s [1]. Despite these declines, infectious diseases are responsible for most refugee deaths [2]. Refugees are particularly vulnerable to infections as a consequence of undernutrition, unclean water, poor sanitation, over-crowding and limited access to health care. Control of communicable diseases is especially important in refugee camps as these environments may foster the re-emergence of previously controlled diseases, particularly when compounded by poor surveillance, monitoring and response. With the average refugee camp operational for more than five years, camp management strategies must consider long-term approaches to providing adequate health services, especially prevention and treatment of infectious diseases.

With almost two-thirds (63%) of the world's refugees, internally displaced persons (IDPs), returnees and other persons of concern (PoCs) to the United Nations High Commissioner for Refugees (UNHCR) living in malaria endemic regions, malaria remains a significant threat to the health of refugee populations, particularly in sub-Saharan Africa [3]. Women of reproductive age and children constitute a majority of refugee populations, and pregnant women and children are at greatest risk for severe malaria and death [4]. Migration from regions of low to high malaria endemicity heightens malaria risk in susceptible refugee populations [5]. Conversely, influxes of refugee populations from regions of high to low endemicity may result in malaria transmission to susceptible host country populations if suitable vectors are present.

During the past decade substantial progress has been made to increase access to malaria prevention strategies and effective antimalarial therapy, particularly in sub-Saharan Africa [6]. Use of insecticide-treated nets (ITNs), intermittent preventive treatment in pregnancy (IPTp), rapid diagnostic tests (RDTs) and artemisinin-based combination therapies (ACTs) have resulted in marked reductions in malaria incidence and mortality in many malaria endemic regions. Interventions specific to refugee camp settings, including insecticide-treated shelters and plastic sheeting, also reduce the burden of disease due to malaria [7,8].

The UNHCR's Strategic Plan for Malaria Control (2008-12) includes support and promotion of malaria control policies and programs to reduce morbidity and mortality [9]. The key approaches are provision of internationally accepted malaria control services to refugees and other PoCs during emergencies and appropriate protection against malaria for vulnerable and at risk populations. These strategies aim to prevent malaria in pregnant women and children through the distribution of long-lasting insecticide-treated nets (LLITNs), provision of IPTp, and the use of accurate diagnostic tests and effective treatment for persons with malaria.

In 2009, UNHCR was responsible for protecting 10.5 million refugees forcibly displaced by conflict. The majority of these refugees resided in Asia, but more than 20% lived in Africa. UNHCR also provides protection to over half of the estimated 26 million IDPs worldwide. To improve the quality and consistency of health information in protracted refugee situations, UNHCR introduced a health information system (HIS) in 2006. The HIS was first piloted in three countries in East Africa (Tanzania, Kenya and Ethiopia) and is currently operational in 85 refugee sites in 16 countries, monitoring health services provided to more than 1.5 million site-based refugees. We report malaria incidence, morbidity and mortality using UNHCR's HIS data from 60 refugee sites in nine countries with at least 50 cases of malaria per 1,000 refugees.

## Methods

UNHCR's HIS was designed to monitor primary health care with the aim of improving refugee health. The HIS is based on guidelines that describe data collection, management and analysis procedures [10]. A comprehensive description of case definitions, the data needed for calculation of each indicator, measurement guidelines, and reporting formats are provided in a toolkit [11]. Weekly tally sheets from each health care facility are converted to numerical totals in a standardized reporting format and submitted to the site health manager. Weekly reports are aggregated into monthly reports submitted to the supervising health agency, usually a non-governmental organization (NGO) partner. Monthly reports are transferred by health agency staff to computerized reporting forms submitted to the local UNHCR sub-office where they are uploaded into the HIS and made accessible to UNHCR branch offices and headquarters. Indicators used by the HIS to measure malaria control activities include total and under-5 incidence of both suspected and confirmed malaria; the proportion of malaria cases confirmed by diagnostic tests; total and under-5 proportionate morbidity due to malaria; total and under-five malaria mortality by sex; total and under-5 proportionate mortality due to malaria; percentage of pregnant women receiving an LLITN or ITN during pregnancy; and the percentage of pregnant women presenting at antenatal care who received two doses of sulfadoxine-pyrimethamine (SP) for IPTp. Suspected cases of malaria were diagnosed based on clinical signs and symptoms while confirmed cases of malaria were positive by RDT or microscopy, although camps differed in the use of confirmatory diagnostic tests. Deaths attributable to malaria were based on oral reports.

Data on malaria incidence, mortality and case fatality were analyzed for three countries with data available between January 2006 and December 2007 (Ethiopia, Kenya and Tanzania) and nine countries with data available between January 2008 and December 2009 (Burundi, Chad, Cameroon, Ethiopia, Kenya, Sudan, Tanzania, Thailand, and Uganda). Although the HIS was piloted in 2006, data from 2006 and 2007 were comprehensively collected in UNHCR camps from only Ethiopia, Kenya and Tanzania. Data were analyzed at the camp and country levels, and sites were eligible to be included in country-level analysis if monthly reports were available for eight months or more per year. Countries were eligible for analysis only if at least 70% of sites had available data. The analysis was restricted to those countries with an annual malaria incidence of at least 50 cases of suspected and confirmed malaria per 1000 refugees as malaria incidence rates below this threshold were unstable. Thus, the primary analysis included 2008-09 HIS data from UNHCR sites in nine countries: Burundi, Chad, Cameroon (2009 only), Ethiopia, Kenya, Sudan, Tanzania, Thailand, and Uganda.

Not all camps in each country had data for each month within the study period as camps were incorporated into the HIS at different times, camps may have opened or closed during this period, or data may not have been reported. Camp populations also varied, sometimes dramatically due to refugee influx or camp closures. To adjust for these sources of variability, we aggregated camps by country and performed country-level analyses. We also aggregated monthly counts of malaria morbidity and mortality from 2008-2009 into an annual rate averaged over this two-year period. This annual rate was calculated by dividing the average annual number of reported cases or deaths per camp over the two-year period by the overall average refugee population. Two approaches were used to estimate malaria incidence, based on confirmed and suspected cases of malaria (the latter diagnosed by clinical signs and symptoms). The sum of suspected and confirmed cases provided an upper limit on the total possible number of malaria cases seeking care, while the incidence rate based solely on confirmed cases provided a lower limit on the total possible number of cases.

Trends in incidence at both the country and site levels were analyzed using annual incidence rates. For this analysis, data from 2006 and 2007 were included if they met the eligibility criteria, i.e. monthly reports were available for at least eight months and at least 70% of sites had available data. Only Ethiopia, Kenya, and Tanzania had data available from 2006-2009, but all countries except Cameroon were included in this analysis.

Average annual malaria mortality rates for 2008-2009 for children younger than five years of age were mapped by UNHCR site and overlaid on estimated malaria endemicity based on *P. falciparum *parasite prevalence generated by the Malaria Atlas Project [12] to identify geographical trends and comparisons with expected rates. Average annual malaria case fatality ratios (CFR) were calculated based on the total number of deaths attributed to malaria divided by the number of confirmed cases in 2008 and 2009, averaged over the two-year period.

Poisson regression was used to determine whether LLITN (or ITN) and IPTp coverage rates for pregnant women, as markers of malaria control interventions, were associated with malaria incidence in children younger than five years of age, accounting for clustering by site. This analysis included monthly data reported from sites through December 2009. Incidence was defined as the sum of confirmed and suspected cases per 1,000 children per month. IPTp coverage for pregnant women was defined as the number of women who received two doses of sulphadoxine-pyrimethamine (SP) at the time of delivery divided by the number of live births, and LLITN (or ITN) coverage was defined as the number of nets provided during antenatal visits divided by the number of live births. Stata (release 10.1, StataCorp) software was used for analysis.

## Results

### Study Populations

An average of 1,178,888 refugees resided in 60 refugee sites over the study period 2008-2009, with a median population of 16,544 refugees per site (interquartile range [IQR]: 8,076, 21,542). Children younger than five years of age comprised 16.3% of all refugees (N = 192,238), half of whom were girls (50.2%). UNHCR camps in Kenya and Chad had refugee populations in excess of 30,000 children younger than five years of age, sites in Ethiopia, Thailand, Uganda and Tanzania had refugee populations between 12,000 and 30,000 children, and sites in Cameroon, Burundi and Sudan had the smallest refugee population, each with fewer than 10,000 children.

### Malaria Incidence

Between 2008 and 2009, an annual average of 111,571 confirmed cases of malaria were reported, of which 40,410 (36.2%) were in children younger than five years of age. Annual average malaria incidence rates based on confirmed cases were 95 cases per 1,000 persons among all refugees and 210 cases per 1,000 children younger than five years of age. The highest incidence of malaria was in camps in Tanzania (Table 1), where the annual incidence of malaria was 399 confirmed cases per 1,000 refugees and 728 cases of confirmed malaria per 1,000 refugee children younger than five years. UNCHR camps in Kenya had the lowest annual malaria incidence rates of 21 and 26 confirmed cases per 1,000 refugees and refugee children younger than five years, respectively (Table 1).

**Table 1:** Annual average malaria incidence among refugees, 2008-2009

	Malaria Cases and Incidence Rates (cases per 1,000 persons per year)					
	Confirmed Malaria		Suspected Malaria		Highest Potential Malaria Rate (Confirmed + Suspected)	
	N	Incidence Rate (95% CI)	N	Incidence Rate (95% CI)	N	Incidence Rate (95% CI)
***Total Population***						
Burundi	1,182	68 (64-71)	3,019	173 (167-178)	4,201	240 (234-247)
Cameroon	618	171 (159-184)	1,415	392 (376-409)	2,033	564 (547-580)
Chad	16,216	63 (62-64)	14,502	56 (55-57)	30,718	119 (118-121)
Ethiopia	1,988	30 (29-32)	3,505	53 (52-55)	5,493	84 (81-86)
Kenya	6,179	21 (21-22)	24,196	83 (82-84)	30,375	105 (104-106)
Sudan	7,214	73 (71-75)	19,873	201 (199-204)	27,086	274 (272-277)
Tanzania	58,430	399 (397-402)	16,575	113 (112-115)	75,004	513 (510-515)
Thailand	10,309	70 (68-71)	15	0 (0-0)	10,324	70 (68-71)
Uganda	9,438	62 (61-63)	66,259	437 (434-439)	75,696	499 (496-501)
**Total**	**111,571**	**95 (94-95)**	**149,357**	**127 (126-127)**	**260,928**	**221 (221-222)**
***Children Younger than Five Years of Age***						
Burundi	482	138 (127-150)	1,273	364 (348-380)	1,755	502 (485-519)
Cameroon	181	281 (247-318)	307	477 (438-517)	487	757 (722-790)
Chad	9,111	193 (190-197)	4,696	100 (96-102)	13,807	293 (289-297)
Ethiopia	609	49 (45-53)	1,382	111 (105-116)	1,990	160 (153-166)
Kenya	1,177	26 (24-27)	15,158	332 (328-337)	16,335	358 (354-362)
Sudan	2,474	272 (263-281)	5,191	570 (560-580)	7,665	842 (834-849)
Tanzania	21,225	728 (723-733)	9,946	341 (336-347)	31,170	1,069 (N/A)
Thailand	897	49 (46-52)	1	0 (0-0)	898	49 (47-52)
Uganda	4,256	163 (158-167)	24,805	948 (945-951)	29,061	1,111 (N/A)
**Total**	**40,410**	**210 (208-212)**	**62,756**	**326 (324-329)**	**103,166**	**537 (534-539)**

Overall, 43% of malaria cases were confirmed by laboratory tests (Table 1). Using an incidence rate based on the sum of confirmed and suspected cases, UNHCR sites in Cameroon, Tanzania, and Uganda had the highest annual malaria incidence (Table 1) ranging from 499 to 564 cases per 1,000 refugees, and 757 to 1,111 cases per 1,000 refugee children younger than five years.

**Figure 1:**
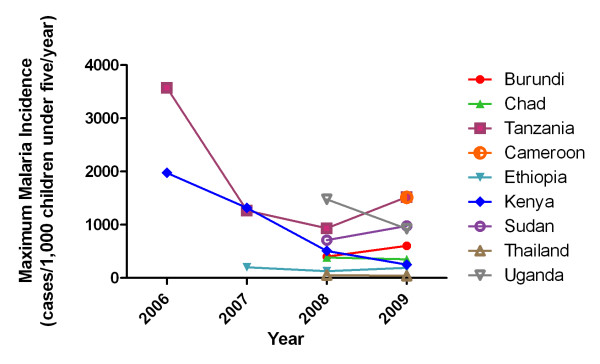
**Annual incidence of malaria among refugee children younger than five years of age in UNHCR sites in selected countries**.

Annual malaria incidence data at the country level from 2006 to 2009 were analyzed for temporal trends, although only Kenya and Tanzania had data available beginning in 2006 (Figure 1). Malaria incidence in children younger than five years of age, based on the sum of confirmed and suspected cases, decreased between the first and last year of reporting for sites in Kenya (-87.3%), Tanzania (-57.5%), Uganda (-37.4), Chad (-9.3%) and Ethiopia (-5.7%). However, an increase in incidence between 2008 and 2009 was observed at sites in Burundi (+50.3%) and Sudan (+37.4%). Overall, there was a slight increase in malaria incidence between 2008 and 2009 in four of the seven countries with available data. The proportion of confirmed cases generally increased as RDTs become more widely used, but this increase was not consistent across all countries (data not shown).

### Malaria Mortality

The annual, all-cause mortality rate in UNHCR sites was 2.8 per 1,000 refugees with an annual malaria mortality rate of 0.3 per 1,000 refugees in 2008-09 (Table 2). Malaria accounted for 12.3% of all refugee deaths in the selected countries, with a range of 0.9% to 24.8%. Annual, all-cause mortality rates were highest in refugee sites in Sudan and Thailand (both with 3.6 deaths per 1,000 refugees) and lowest in UNHCR camps in Ethiopia (1.3 deaths per 1,000 refugees). Annual malaria mortality rates were highest in sites in Sudan (0.9 deaths per 1,000 refugees), Uganda (0.7 deaths per 1,000 refuges) and Tanzania (0.7 deaths per 1,000 refugees). Sites in these countries also reported the highest proportions of deaths attributed to malaria: 24.8%, 24.5%, and 23.0%, respectively. Sites in Ethiopia and Thailand had the lowest annual malaria mortality rates (Table 2) and the lowest proportions of deaths attributed to malaria (2.4% in Ethiopia and 0.9% in Thailand). The proportion of malaria deaths that occurred in health care facilities ranged from 33% (one camp in Burundi, 13 camps in Chad) to 100% (two camps in Ethiopia).

**Table 2:** Annual average malaria mortality among refugees, 2008-2009

	Malaria Mortality					
	Crude Mortality		Malaria Mortality		Malaria Case Fatality Ratio** (95% CI)	Deaths Attributed to Malaria (percent, 95% CI)
	N	Mortality Rate (95% CI)*	n	Mortality Rate (95% CI)*		
***Total Population***						
Burundi	44	2.5 (1.8-3.4)	5	0.3 (0.0-0.7)	4.2 (1.4-9.8)	11% (4-25)
Cameroon	9	2.5 (1.1-4.7)	1	0.3 (0.0-1.5)	1.6 (0.0-9.0)	11% (3-48)
Chad	762	3.0 (2.8-3.2)	43	0.2 (0.1-0.2)	2.7 (1.9-3.6)	6% (4-8)
Ethiopia	85	1.3 (1.0-1.6)	2	0.0 (0.0-0.1)	1.0 (0.1-3.6)	2% (0-8)
Kenya	655	2.3 (2.1-2.4)	54	0.2 (0.1-0.2)	8.7 (6.6-11.4)	8% (6-11)
Sudan	355	3.6 (3.2-4.0)	87	0.9 (0.7-1.1)	12.1 (9.7-14.9)	25% (20-29)
Tanzania	430	2.9 (2.7-3.2)	99	0.7 (0.6-0.8)	1.7 (1.4-2.1)	23% (19-27)
Thailand	527	3.6 (3.3-3.9)	5	0.0 (0.0-0.1)	0.4 (0.2-1.1)	1% (0-2)
Uganda	453	3.0 (2.7-3.3)	113	0.7 (0.6-0.9)	11.9 (9.9-14.4)	25% (21-29)
**Total**	**3,319**	**2.8 (2.7-2.9)**	**408**	**0.3 (0.3-0.4)**	**3.7 (3.3-4.0)**	**12% (11-13)**
***Children Under Five Years of Age***						
Burundi	21	6.0 (3.7-9.2)	4	1.1 (0.3-2.9)	8.3 (2.3-21.1)	19% (5-42)
Cameroon	3	4.7 (0.9-13.6)	1	1.6 (0.0-8.6)	5.5 (0.1-30.4)	33% (1-91)
Chad	404	8.6 (7.8-9.4)	33	0.7 (0.5-1.0)	3.6 (2.5-5.1)	8% (6-11)
Ethiopia	34	2.7 (1.9-3.8)	1	0.0 (0.0-0.4)	1.6 (0.0-9.1)	3% (0-15)
Kenya	307	6.7 (6.0-7.5)	30	0.7 (0.4-0.9)	25.5 (17.3-36.2)	10% (7-14)
Sudan	144	15.8 (13.4-18.6)	37	4.1 (2.9-5.6)	15.0 (10.6-20.6)	26% (19-34)
Tanzania	231	7.9 (6.9-9.0)	59	2.0 (1.5-2.6)	2.8 (2.1-3.6)	26% (20-32)
Thailand	104	5.6 (4.6-6.8)	0	0.0 (0.0-0.2)	0.0 (0.0-4.1)	0% (0-4)
Uganda	219	8.4 (7.3-9.5)	67	2.6 (2.0-3.3)	15.7 (12.2-20.0)	31% (25-37)
**Total**	**1,465**	**7.6 (7.2-8.0)**	**231**	**1.2 (1.1-1.4)**	**5.7 (5.0-6.5)**	**16% (14-18)**

*annual mortality rates expressed as deaths per 1000 persons

** case fatality rates expressed as deaths per 1000 confirmed cases

**Figure 2:**
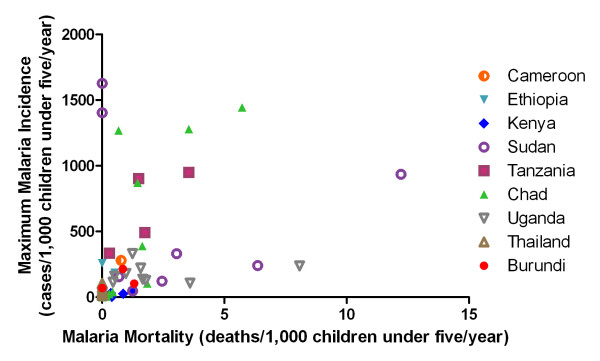
**Annual malaria incidence and mortality by camp among refugee children younger than five years of age, averaged over 2008-2009**.

For children younger than five years of age, the annual all-cause mortality rate was 7.6 per 1,000 refugee children and the annual malaria mortality rate was 1.2 per 1,000 refugee children (Table 2). Malaria was the cause of 16% of deaths in refugee children younger than five years of age, with a range of 0% to 33%. Crude annual mortality rates in children younger than five years of age were highest in UNHCR sites in Sudan (15.8/1,000 children) and lowest in Ethiopia (2.6/1,000 children). Annual malaria mortality rates were highest in sites in Sudan (4.1/1,000 children; accounting for 26% of under-five deaths) and Uganda (2.6/1,000 children, accounting for 31% of under-five deaths). There was no clear relationship between malaria incidence and mortality in children younger than five years of age (Figure 2).

**Figure 3:**
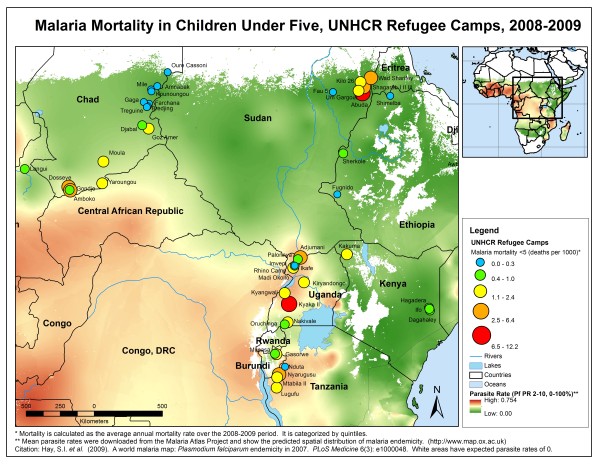
**Malaria mortality among refugee children by camp for 2008-2009, mapped on malaria endemicity derived from the Malaria Atlas Project**.

Despite close proximity and similar levels of malaria endemicity, malaria mortality rates in children varied greatly between some neighboring UNHCR sites (Figure 3). In Chad, for example, most camps in the northeast had low malaria mortality rates among refugee children consistent with the low estimated parasite prevalence. However, three refugee camps in southwestern Chad had varying malaria mortality rates despite geographic proximity and an estimated parasite prevalence of 35%. Dosseye had the smallest camp population (8,147 refugees averaged over 2008-2009) but the highest malaria mortality rate among camps in Chad (5.7 deaths per 1000 children). Amboko and Gondje were larger camps (11,996 and 14,692 refugees averaged over 2008-2009, respectively) and had lower malaria mortality rates in children (3.5 and 0.7 deaths per 1,000 children, respectively). However, these three camps had similar malaria incidence rates in children younger than five years of age, among the highest recorded of all sites, ranging from 1,267 confirmed cases/1,000 children in Gondje to 1,442 confirmed cases/1,000 children in Dosseye.

Malaria CFR for children younger than five years of age ranged from 1.6 deaths per 1,000 children with confirmed malaria in UNHCR camps in Ethiopia to 25.5 deaths per 1,000 children with confirmed malaria in UNHCR camps in Kenya (Table 2). Sites in Sudan and Uganda had CFR of 15.0 and 15.7 deaths per 1,000 children, respectively, whereas malaria CFR in sites in Thailand, Ethiopia, Tanzania, Chad, Cameroon and Burundi ranged from 0.0 to 8.8 deaths per 1,000 children.

### Malaria and Pregnancy

Provision of LLITNs (and ITNs) and IPTp with at least two doses of SP to pregnant women were the two malaria control interventions reported in the HIS. The estimated percentage of pregnant women who received LLITNs (or ITNs) ranged from 9.5% in UNHCR camps in Burundi to 98.5% in camps in Tanzania (Table 3). The estimated percentage of pregnant women who received IPTp, using the number of live births as denominator, ranged from 0.7% in Burundi to 99.4% in Tanzania. Monthly IPTp and ITN coverage rates were not significantly associated with malaria incidence in children younger than five years of age (incidence rate ratio [IRR] = 1.00, p < 0.001 and IRR = 1.00, p = 0.631, respectively).

**Table 3:** Annual average coverage rates of LLINs (and ITNs) and IPTp, 2008-2009

Country	Insecticide Treated Nets (ITN) Distribution among Pregnant Women		Presumptive Malaria Treatment (SP) During Pregnancy	
	N	Coverage rate (95% CI)	N	Coverage rate (95% CI)
Burundi	49	9.5% (7.2-12.4)	4	0.7% (0.2-2.0)
Cameroon	56	97.4% (88.1-99.6)	56	97.4% (88.1-00.6)
Chad	4,637	62.1% (60.9-63.1)	5,871	78.6% (77.6-79.5)
Ethiopia	1,435	68.1% (66.1-70.1)	1,349	64.0% (62.0-66.1)
Kenya	2,669	32.3% (31.3-33.3)	7,175	86.7% (86.0-87.5)
Sudan	654	28.3% (26.5-30.2)	1,807	78.3% (76.6-80.0)
Tanzania	5,307	98.5% (98.1-98.8)	5,360	99.4% (99.2-99.6)
Thailand	701	16.1% (15.0-17.2)	62	1.4% (1.1-1.8)
Uganda	2,160	60.4% (58.7-62.0)	2,609	72.9% (71.4-74.3)
**Total**	**17,667**	**51.9% (51.4-52.4)**	**24290**	**71.3% (70.9-71.8)**

*includes only women with live births

## Discussion

UNHCR's HIS data were used to derive malaria incidence and mortality estimates for more than one million refugees living in 60 camps in nine countries, the largest analysis of malaria in post-emergency refugee sites. As expected, annual malaria incidence rates varied geographically and over time. Although few comparable data are available, a retrospective mortality survey from 1998 to 2000 in 51 post-emergency camps in Azerbaijan, Ethiopia, Myanmar, Nepal, Tanzania, Thailand and Uganda reported a higher overall incidence of malaria of 48 cases per 1,000 persons per month (range 0-325; approximately 576 cases per 1,000 persons per year) and 78 cases per 1,000 in children younger than five years of age per month (range 0-463; approximately 936 cases per 1,000 children per year) [2]. The more recent UNHCR HIS data show that malaria remains a significant cause of morbidity and mortality among refugees despite declining transmission rates in many regions of sub-Saharan Africa [6].

Progress has been made in reducing the burden of malaria among refugees in some countries. The annual incidence of malaria in children younger than five years of age decreased more than one third in UNHCR sites within Kenya, Tanzania and Uganda between 2006 and 2009, consistent with changes in the burden of malaria in these countries [6]. In contrast, the annual incidence of malaria in children increased slightly in four countries between 2008 and 2009. Although a short time interval to assess trends in malaria incidence, the largest increases in malaria incidence over the last two years of observation occurred at two sites in Ethiopia. One site, Shimelba, is close to sites in Sudan that also had an increase in malaria incidence between 2008 and 2009. Sudan had the highest malaria mortality rate among refugees younger than five years of age (4.1 deaths per 1000 refugee children) similar to the estimated malaria mortality rate of non-refugee Sudanese children (4.6 deaths per 1,000 children per year) [13].

Data on malaria control interventions, specifically IPTp and ITN coverage, were not associated with malaria incidence in children younger than five years of age. However, changes in UNHCR's policies since 2006 likely contributed to declines in malaria incidence among refugees. Providing LLITNs has been one of UNHCR's primary prevention strategies. At a cost of $5 per net, UNHCR has spent approximately $10,000,000 between 2005 and 2008 on LLITN procurement. In accordance with the Malaria Strategic Plan 2008-12, UNHCR aims to increase LLITN coverage of vulnerable groups in emergency situations to full coverage in stable settings and, with support from the UN Foundation's Nothing But Nets Campaign, provide one net for every 2 persons to sleep under (3-4 nets per household) in 17 African countries most affected by malaria.

Monitoring LLITN use may further improve malaria control. The monitoring of LLITN distribution at sites near Dadaab, northeast Kenya serves as a model program. LLITN distribution targeted 80,000 people in 2009, including pregnant women, children under the age of five years old, hospitals, chronically ill, and the elderly. Community leaders identified recipients and routine distribution occurred at clinics and hospitals. LLITN ownership was monitored through 2010, nested within nutrition surveys. Further quantitative and qualitative studies were conducted to identify net coverage, condition, maintenance practices, factors that affect usage and net preference. LLITN coverage increased from approximately 60% to 86%. Incorporating data on LLITN coverage and use within nutrition surveys can provide important information for targeted interventions in protracted refugee settings.

The use of RDTs for case diagnosis was implemented in many refugee camps but HIS data indicated that only 43% of malaria cases were confirmed. Thus, while diagnostics were available at most UNHCR sites, high coverage was not achieved during the study period. UNHCR subsequently developed standard operating procedures for confirmation of malaria and is working to achieve high coverage of RDT use in malaria endemic areas.

Since 2008, ACT has been available in malaria-endemic countries in Africa and has reached near universal coverage in UNHCR camps, consistent with WHO recommended malaria treatment guidelines [14]. Shortages of ACT were experienced at camps in Cameroon, Cote d'Ivoire, Kenya, Tanzania, Uganda, and Zimbabwe during the study period, but UNHCR worked closely with the Novartis Foundation to provide drugs to those countries experiencing procurement and distribution challenges.

Including refugees and IDPs in national strategic plans for malaria can decrease morbidity and mortality among displaced persons. In a review of 15 national strategic plans from countries in Africa that host ≥10,000 refugees, only three made specific reference to refugees and five made broad mention of refugees without discussion of specific activities [15]. Governments that signed the 1951 Convention relating to the Status of Refugees have a legal obligation to assist refuges, including the provision of health care. Furthermore, extending malaria control interventions to refugees will be critical to achieving malaria control and elimination within countries with large populations of refugees.

Several limitations in HIS data collection may have biased these findings. Data were aggregated over the two-year period from January 2008 to December 2009 and averaged to determine a mean annual rate. These average annual rates mask differences in malaria incidence, morbidity and mortality between 2008 and 2009. We aggregated camp-level data by countries but heterogeneities in malaria transmission and control exist within countries. Case definitions, reporting practices, and reporting quality varied at the camp and country levels. Perhaps most importantly, accurate diagnosis of malaria and attributing malaria as the cause of death are prone to misclassification. However, given that similar methods were used over the study period, the interpretation of trends should be valid.

## Conclusions

Despite declines in malaria incidence among refugees in several countries, malaria remains a significant cause of mortality, particularly among children younger than five years of age. Further progress in malaria control, both within and outside of post-emergency sites, will be necessary to further reduce malaria incidence and mortality among refugees and achieve global goals in malaria control and elimination.

## List of Abbreviations

ACT: artemisinin-combination therapy; CFR: case fatality ratios; HIS: health information system; IDPs: internally displaced persons; IPTp: intermittent preventive treatment in pregnancy; IQR: interquartile range; ITN: insecticide-treated net; LLITNs: long-lasting insecticide-treated nets; MAP: Malaria Atlas Project; PoCs: persons-of-concern; RDT: rapid diagnostic test for malaria; SP: sulfadoxine-pyrimethamine; UNHCR: United Nations High Commissioner for Refugees

## Competing interests

The authors declare that they have no competing interests.

## Authors' contributions

JA conducted the analyses and drafted the manuscript. SD conceived of the study and participated in the design, coordination and drafting of the manuscript. CH conceived of the study and participated in the design, coordination and drafting of the manuscript. PS conceived of the study and participated in the design, coordination and drafting of the manuscript. WJM participated in the design, coordination and drafting of the manuscript. All authors read and approved the final manuscript.

## References

[B1] Salama P Spiegel P Talley L Waldman R Lessons learned from complex emergencies over past decade Lancet 2004 364 1801 1813 10.1016/S0140-6736(04)17405-9 15541455

[B2] Spiegel P Sheik M Gotway-Crawford C Salama P Health programmes and policies associated with decreased mortality in displaced people in postemergency phase camps: a retrospective study Lancet 2002 360 1927 1934 10.1016/S0140-6736(02)11915-5 12493259

[B3] Connolly MA Gayer M Ryan MJ Salama P Spiegel P Heymann DL Communicable diseases in complex emergencies: impact and challenges Lancet 2004 364 1974 1983 10.1016/S0140-6736(04)17481-3 15567014

[B4] Rowland M Nosten F Malaria epidemiology and control in refugee camps and complex emergencies Ann Trop Med Parasitol 2001 95 741 754 10.1080/00034980120103405 11784429

[B5] Bloland PB Williams HA Malaria Control During Mass Population Movements and Natural Disasters 2003 Washington, DC: The National Academies Press 25057635

[B6] O'Meara WP Mangeni JN Steketee R Greenwood B Changes in the burden of malaria in sub-Saharan Africa Lancet Infect Dis 2010 10 545 555 10.1016/S1473-3099(10)70096-7 20637696

[B7] Graham K Mohammad N Rehman H Nazari A Ahmad M Kamal M Skovmand O Guillet P Allan R Zaim M Insecticide-treated plastic tarpaulins for control of malaria vectors in refugee camps Med Vet Entomol 2002 16 404 408 10.1046/j.1365-2915.2002.00395.x 12510893

[B8] Graham K Rehman H Ahmad M Kamal M Khan I Rowland M Tents pre-treated with insecticide for malaria control in refugee camps: an entomological evaluation Malar J 2004 3 25 10.1186/1475-2875-3-25 15253773 PMC493276

[B9] UNHCR UNHCR's Strategic Plan for Malaria Control 2008-2012 2008 Geneva: UNHCR21929774

[B10] UNHCR Health Information System Training Manual to Support Implementation in Refugee Operations 2007 Geneva: UNHCR

[B11] UNHCR Health Information System Tool Kit 2010

[B12] Hay SI Guerra CA Gething PW Patil AP Tatem AJ Noor AM Kabaria CW Manh BH Elyazar IR Brooker S A world malaria map: *Plasmodium falciparum *endemicity in 2007 PLoS Med 2009 6 e1000048 19323591 10.1371/journal.pmed.1000048 PMC2659708

[B13] World Health Organization World Malaria Report 2008 Geneva, WHO

[B14] Eastman RT Fidock DA Artemisinin-based combination therapies: a vital tool in efforts to eliminate malaria Nat Rev Microbiol 2009 7 874 10.1038/nrmicro2239 PMC2901398 19881520

[B15] Spiegel PB Hering H Paik E Schilperoord M Conflict-affected displaced persons need to benefit more from HIV and malaria national strategic plans and Global Fund grants Confl Health 2010 4 2 10.1186/1752-1505-4-2 20205901 PMC2827465

